# Academic achievement prediction in higher education through interpretable modeling

**DOI:** 10.1371/journal.pone.0309838

**Published:** 2024-09-05

**Authors:** Sixuan Wang, Bin Luo

**Affiliations:** School of Foreign Languages, Wuhan Business University, Wuhan, Hubei, People’s Republic of China; Abdul Wali Khan University Mardan, PAKISTAN

## Abstract

Student academic achievement is an important indicator for evaluating the quality of education, especially, the achievement prediction empowers educators in tailoring their instructional approaches, thereby fostering advancements in both student performance and the overall educational quality. However, extracting valuable insights from vast educational data to develop effective strategies for evaluating student performance remains a significant challenge for higher education institutions. Traditional machine learning (ML) algorithms often struggle to clearly delineate the interplay between the factors that influence academic success and the resulting grades. To address these challenges, this paper introduces the XGB-SHAP model, a novel approach for predicting student achievement that combines Extreme Gradient Boosting (XGBoost) with SHapley Additive exPlanations (SHAP). The model was applied to a dataset from a public university in Wuhan, encompassing the academic records of 87 students who were enrolled in a Japanese course between September 2021 and June 2023. The findings indicate the model excels in accuracy, achieving a Mean absolute error (MAE) of approximately 6 and an R-squared value near 0.82, surpassing three other ML models. The model further uncovers how different instructional modes influence the factors that contribute to student achievement. This insight supports the need for a customized approach to feature selection that aligns with the specific characteristics of each teaching mode. Furthermore, the model highlights the importance of incorporating self-directed learning skills into student-related indicators when predicting academic performance.

## Introduction

### Context and motivation

Academic achievement is of paramount importance in educational contexts, serving as a key indicator of both learning ability and the effectiveness of school administration and teaching standards [1]. The prediction of academic achievement is a continuously evolving topic in educational management. The integration of predictive models in education empowers educators to make well-informed choices, offer specific support, and enhance teaching strategies, thereby improving student learning outcomes [2].

Previous research on achievement prediction primarily utilized statistical analysis methods to process data and forecast outcomes, with data mainly derived from educational management systems, student identification cards, or surveys [3]. ML techniques, known for their ability to tackle complex, nonlinear problems without presuppositions, are adept at identifying connections between various parameters [4]. The state-of-the-art ML techniques for prediction [5] include K-Nearest Neighbors (KNN), Decision Trees, Random Forests (RF), Support Vector Machines (SVM), Neural Networks, and Naive Bayes. Recent scholarly efforts, both domestically and internationally, have been geared towards increasing the precision of student achievement predictions through technological innovations in algorithms [6–8].

Despite these developments, challenges remain in the domain of achievement prediction. A primary issue is the limited alignment between the outcomes produced by ML algorithms and the foundational principles of education and instruction, leading to hesitancy among educators in relying on these models. Additionally, there is a gap in thorough data analysis, examination of relationships, and investigation into variables that impact student academic performance patterns.

### Contribution of the study

In addressing these challenges, our study delivers distinctive contributions to the field of interpretable machine learning within the context of higher education. We delineate these contributions as follows:

- Theoretical contribution: this study introduces ML models coupled with game theory-based SHAP analysis which aims to develop and validate the XGB-SHAP model, a novel approach for interpreting machine learning-based predictions of student achievement, and explore its efficacy across various teaching modalities.
- Practical contributions: It evaluates the significance of different indicators and their positive or negative impacts on prediction outcomes, thus shedding light on the educational implications of achievement prediction models. The findings of this study provide empirical data support for teachers and educators, facilitating the refinement of their instructional strategies.
- Comparative analysis: It explores student achievement prediction models in three distinct educational settings: online, offline, and blended teachings. This exploration reveals variances in teaching patterns across these modes, yielding practical advice for educators in applying these prediction models.

### Structure of the article

This paper is organized as follows: Section ‘Literature review’ presents a review of related literatures, providing a comprehensive review of the existing literature on student achievement prediction, examines the prevailing issues and identifies the gaps within the current body of research. Section ‘Methodology’ details the methodology employed in this study, introduces the interpretable performance prediction framework and the indicators system used in this paper and outlines the methodology used to conduct the data analysis for this paper. The findings and their implications are discussed in Sections ‘Case study’ and ‘Results’ respectively. The paper concludes with a summary of our key findings in the final Section ‘Discussion and Conclusions’. Table 1 illustrates the list of abbreviations.

**Table 1:** List of abbreviations.

Abbreviation	Full form
ML	Machine Learning
SHAP	SHapley Additive exPlanations
XGBoost	Extreme Gradient Boosting
MAE	Mean Absolute Error
RF	Random Forest
SVM	Support Vector Machines
AI	Artificial Intelligence
BPNN	Backpropagation Neural Network

## Literature review

### Previous research

#### Student achievement prediction indicators

Prediction accuracy largely depends on the careful selection of indicators. The initial and most critical step is the selection of appropriate input data. Previous research has identified three key groups of student-related features as pertinent input parameters: historical student performance, student engagement, and demographic data (Tomasevic et al., 2020).

Historical student performance has been consistently identified as a reliable predictor. For instance, DeBerard et al. [9] demonstrated that high school GPA is a strong predictor of college academic success. Similarly, Shaw et al. [10] found that combined SAT scores explain about 28% of the variance in first-year college GPA. Moreover, test scores have been used to predict future academic performance in various studies [11].

Regarding student engagement, a notable correlation with academic achievement has been observed [12]. Hussain et al. [13] identified a moderately strong positive correlation between student engagement and academic achievement. With evolving teaching formats like Massive Open Online Courses and the flipped classrooms, several studies have developed predictive models by analyzing student behaviors in learning management systems, such as video interactions, assignment submissions, and forum discussions [14]. With the innovation of modern educational technology tools, including artificial intelligence tools (such as ChatGPT) and virtual reality, significant roles have been played in enhancing student learning outcomes by integrating with educational theories like constructivism, experiential learning, and collaborative learning. These technologies, by offering immersive and interactive learning experiences, have increased student engagement, motivation, and critical thinking skills, thereby positively impacting academic performance [15, 16].

Studies have also considered demographic factors. Research indicates that demographic factors play a moderate role in predictive accuracy, with relevance around 60% in some studies, while others suggest that these variables have a limited impact on prediction precision [5, 17]. Additional indicators, such as student collaboration, teacher-student communication, and psychological factors like motivation and attitude, have also been explored. Recent studies emphasize the importance of considering learners’ psychological well-being and cognitive processes in educational settings [18, 19].These motivational and coping strategies remarkably influence students’ learning approaches and overall educational outcomes [20].

The above discussion shows that student achievement is a composite of cognitive, behavioral, skill-based, and emotional outcomes derived from educational experiences [21]. Although there is a consensus on the selection of certain important indicators, the selection of the dataset for student achievement prediction varies from study to study. Selecting the most suitable dataset depends largely on the specific goals and objectives of the researchers, with no universally accepted guidelines.

#### Student achievement prediction models

Originally, conventional statistical methods such as Discriminant Analysis and Multiple Linear Regression were the predominant approaches in the early stages of educational research [22]. Furthermore, Structural Equation Modeling (SEM) has been widely adopted in the social sciences. However, these traditional methods have often fallen short of delivering consistent and precise predictions or classifications [23].

Recently, an array of machine learning algorithms has been employed, including Multiple Regression, Probabilistic and Logistic Regression, Neural Networks, Decision Trees, Random Forests (RF), Genetic Algorithms, and Bayesian algorithms. These have shown varied levels of success in achieving high predictive accuracy [24]. Comparative studies of machine learning methods have been conducted, with Caruana et al. [25] exploring the performance evaluation of these models. Their research underscores a fundamental point: no single model or method universally excels across all problems and metrics. Tomasevic et al. [5] used the Open University Learning Analytics Dataset for a regression problem, finding that Artificial Neural Networks (ANN) and Decision Trees were the most effective, while KNN, SVM, and Bayesian linear regression were less successful.

While previous approaches using machine learning models for predicting student achievement have focused on model optimization [26], there are growing concerns regarding the opaque nature of complex models, which may hinder their broader application [27].

#### Interpretable machine learning models

Nowadays, with the rapid development of artificial intelligence (AI) technology, ML models are being applied in many critical fields, such as education [28, 29], healthcare [30–32]. However, as the number of parameters soars, the ’black-box’ nature of neural networks has raised concerns. Interpretable machine learning is a promising tool to alleviate concerns regarding the opacity of machine learning models. It equips ML models with the capability to articulate their processes in a manner comprehensible to humans [33].

Broadly, interpretable machine learning methods are divided into two categories: self-interpretation models and post-hoc interpretation methods [34]. Self-interpreting models typically have a simpler structure and include Linear models, Logistic Regression, and Decision Trees. Post-hoc interpretation methods involve either model-independent or model-specific techniques, applicable to various models but may require additional computational resources and analytical expertise.

Post-hoc or model-independent interpretation methods are extensively used in different scenarios. These include Partial Dependence Plot [35], Individual Conditional Expectation [36], Permutation Feature Importance [37], Local Interpretable Model-agnostic Explanations, and the SHAP method. The survey in the field of information resource management revealed that 83.7% of explainable ML applications utilize post-hoc explanation methods, with SHAP (51.2%) and feature importance analysis (34.1%) being the most common. Unlike traditional feature importance which indicates the significance of features without clarifying their impact on predictions, SHAP offers detailed explanations on both sample and feature levels through various visualizations like waterfall diagrams and feature dependency diagrams.

These interpretative approaches have been applied in diverse fields such as medicine, policymaking, and science, aiding in auditing predictions under circumstances like regulatory pressures and the pursuit of fairness [35]. However, the critical aspect of interpretability in machine learning models within the domain of educational management research remains underexplored.

### Research gap

Given the aforementioned limitations, the interpretability of ML is a contentious issue. The various ML algorithms employed often fail to effectively elucidate the relationship between factors influencing students’ academic performance and their grades. Additionally, they struggle to quantify the impact of each feature on the target value and to determine the positive or negative influence of each characteristic. To address these gaps in the literature, our study delves into the following areas:

- Feature Importance Analysis: Our research will quantify the influence of each feature on the prediction of student performance. This involves a detailed examination of the weight and significance of various factors in determining academic outcomes.
- Impact Assessment: We will assess the positive or negative impact of each feature on the target variable. This is crucial for understanding not only the magnitude of the influence but also its direction.
- Model Comparison: By comparing the interpretability and performance of different ML models, our study seeks to identify the most effective approaches for student achievement prediction.
- Practical Implications: We will discuss the practical implications of our findings, focusing on how increased interpretability can enhance educational practices and inform policy-making.

Through this comprehensive approach, our study seeks to bridge the gap in the current research by providing a clearer understanding of the mechanisms behind student achievement prediction models and their implications for educational stakeholders.

## Methodology

### Development of an interpretable performance prediction framework

**Fig 1:**
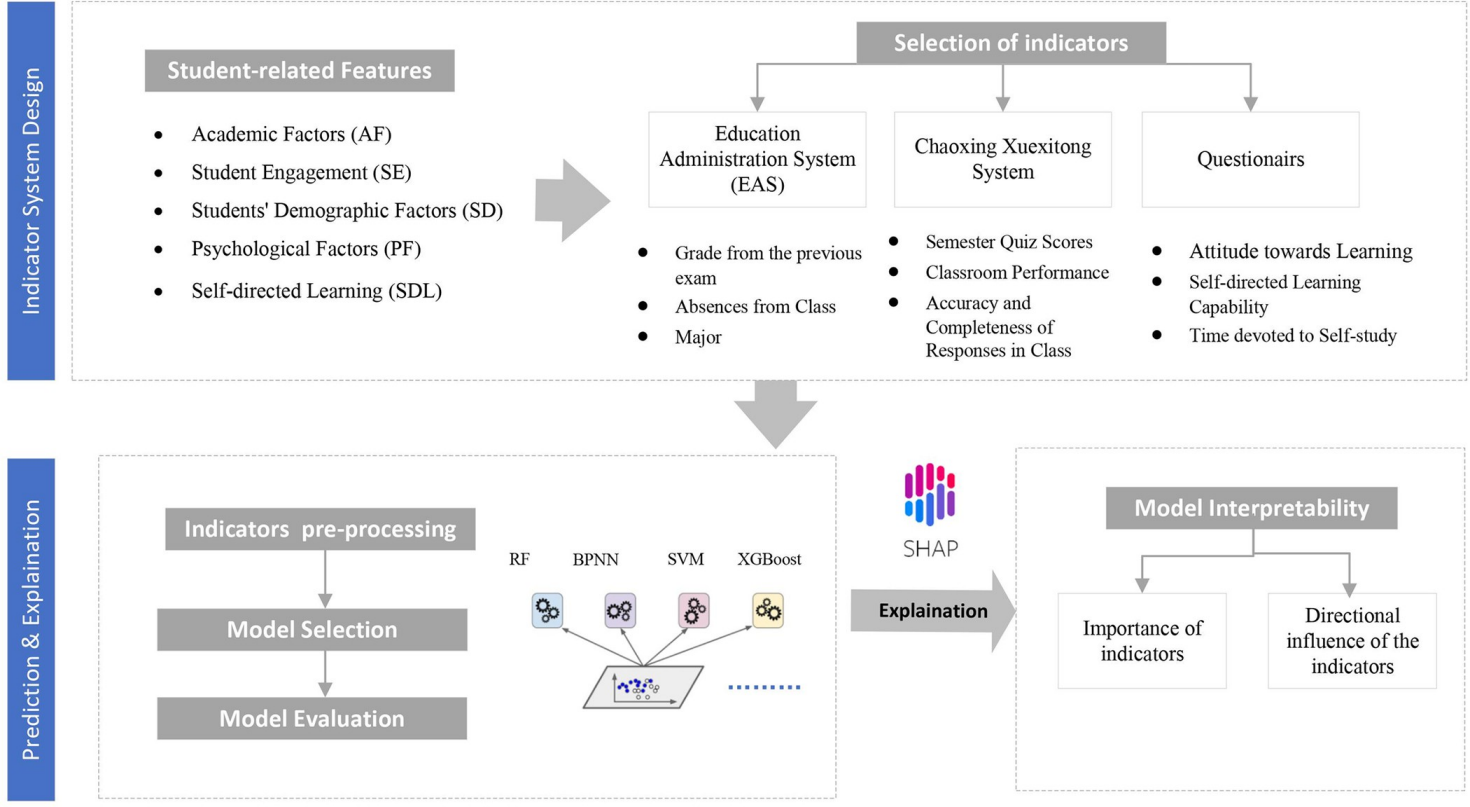
The framework of the interpretable academic achievement prediction model.

As shown in Fig 1, we have developed an interpretable framework for performance prediction. The framework’s core involves extracting five key features: academic factors, student engagement, demographic factors, psychological aspects, and self-directed learning abilities. These features form an input vector that accurately represents factors relevant to achievement prediction. The data for this study is sourced from three main systems: the Education Administration System (EAS), the Chaoxing Xuexitong System, and various questionnaires.

The methodology progresses in three phases. The initial phase involves creating an indicator system from these features. In the subsequent phase, we focus on constructing and elucidating performance prediction models. Four different ML algorithms are applied to our “learning” dataset. Their effectiveness is evaluated using two standard ML metrics: Mean Absolute Error (MAE) and R-squared (*R*^*2*^). The optimal model is then selected based on these evaluations. The final stage of our methodology is the model interpretability phase, which accounts for the educational significance of the model by analyzing the importance and directional influence of the indicators. This phase aims to provide educators with insights to refine their teaching strategies.

### Development of the indicator system

As mentioned in ‘Literature review’ section, prior research insights advocate categorizing student-related features into historical student performance, engagement, and demographic data [5]. To capture a holistic view of learner characteristics, we have expanded this system to include psychological factors and self-directed learning capabilities to form a student achievement prediction indicator system, as shown in Table 2. Considering the minimal variation in age, gender, and other demographic factors in our case study, we have chosen to focus solely on the major as the demographic data point.

**Table 2:** Student achievement prediction indicator system.

Feature	Definition	Indicator
Academic Factors (AF)	Quantifies the academic performance of students, such as exam scores, GPA, or class rank.	1. Grade from the previous exam 2. Semester Quiz Scores
Student Engagement (SE)	Measures the level of student involvement and participation in educational activities, including class attendance, participation in discussions, completion of assignments, and extracurricular involvement.	3. Classroom Performance 4. Accuracy and Completeness of Responses in Class 5. Absences from Class
Psychological Factors (PF)	Focus on students’ psychological characteristics and attributes that contribute to their academic success. They may include variables such as motivation, self-efficacy, self-regulation, learning styles, and attitudes toward learning.	6. Attitude towards Learning
Students’ Demographic Factors (SD)	Recognizes the influence of students’ backgrounds and contexts on their academic outcomes. This data includes variables such as age, gender, specialties or areas of study, and location.	7. Major
Self-directed Learning (SDL)	It refers to the process in which individuals take the initiative and responsibility for their own learning, without relying on external direction or instruction.	8. Self-directed Learning capability 9. Time devoted to self-study

Utilizing practical data from real-world scenarios, our model integrates these nine indicators to predict student achievement. The indicators collectively form an input vector matrix for predicting a student’s academic performance, which can be defined as

$$x(i)={\left[x_{i,1},x_{i,2},x_{i,3},\ldots \ldots ,x_{i,k}\right]}^{T},$$ (1)

where *i* indicates the student’s serial number and *k* the *kth* indicator. The output set *y*_*i*_ represents the student’s final exam score for the current period.

### Model training

As SHAP is a model-agnostic interpretation framework, which enables it to be applied across a spectrum of common predictive models. This versatility allows SHAP to provide insights into the decision-making process of these models by quantifying the contribution of each feature to the prediction, thereby enhancing our understanding of the model’s behavior regardless of its underlying structure or algorithmic approach. Commonly used ML models for academic achievement prediction include RF, BPNN, SVM, and XGboost. The rationale for selecting these four models is their proficiency as data-driven prediction methods. RF, an ensemble learning technique, amalgamates numerous decision trees, thereby reducing variance relative to individual trees. It is known for its superior average prediction performance. BPNN, a supervised learning algorithm, builds multi-layer neural networks inspired by biological neurons and employs a back-propagation algorithm for training, excelling in handling non-linear relationships and high-dimensional data. SVM has gained recognition for its effectiveness in classification, regression, and time-series prediction. XGBoost, enhancing the Gradient Boosting Decision Tree algorithm, stands out for its accuracy and flexibility.

To evaluate and select the most suitable model, we use MAE and *R*^*2*^ as performance metrics, which can be defined as:

$$\mathrm{MAE}=\frac{1}{n}\sum_{i=1}^{n}|y_{i}-{\hat{y}}_{i}|,$$ (2)

$$R^{2}=1-{\frac{\sum_{i=1}^{n}\left(y_{i}-{\hat{y}}_{i}\right)}{\sum_{i=1}^{n}{\left(y_{i}-\bar{y}\right)}^{2}}}^{2}$$ (3)

where *n* represents the number of samples. MAE measures the average absolute error between predicted and actual values, with a lower MAE indicating superior model performance. *R*^2^ assesses the model’s data fit, where a larger *R*^2^ value generally signifies a better fit. An empirical *R*^2^ value greater than 0.4 is considered indicative of a good fit [38].

In this research, a 5-fold cross-validation approach was implemented to fine-tune the hyperparameter to avoid overfit, optimizing them according to the mean value derived from each test set.

### Model interpretability

Addressing the opaque nature of ML models, our research employs the SHAP method for interpretability. Developed by Lundberg and Lee in 2017 [39], SHAP merges various existing approaches to provide a reliable and intuitive explanation of model predictions. It does so by illustrating how predictions shift when certain variables are omitted. The Python SHAP package (https://github.com/slundberg/shap), enables the calculation of SHAP values for any selected model, and it is extensively utilized due to its versatility.

SHAP is characterized by three fundamental properties: local accuracy (the sum of feature attributions equals the model output), missingness (zero attribution for non-present features), and consistency (no decrease in feature attribution despite an increased marginal contribution). A notable advantage of SHAP is its model-agnostic nature, making it applicable to any machine learning model.

The principle of SHAP can be explained as follows: Assume the *ith* sample is *x*_*i*_, with the *jth* feature of this sample being *x*_*ij*_, and the model’s predicted value for this sample as *y*_*i*_. The baseline value for the model (often the average of the target variable) is *y*_*base*_. The SHAP value then follows the equation:

$$y_{i}=y_{base}+f(x_{i1})+f(x_{i2})+\ldots +f(x_{ik})$$ (4)

where *f*(*x*_*ik*_) is the SHAP value of *x*_*ij*_. Intuitively, *f*(*x*_*ij*_,1) indicates the contribution of the 1st feature in the *ith* sample to the final predicted value *y*_*i*_. A value pf *f*(*x*_*i*_,1) greater than 1 implies that the feature enhances the predicted value, whereas a negative value suggests a diminishing effect.

## Case study

### Datasets

Data for this study was obtained from the EAS of a Wuhan-based public university. This system provided access to students’ personal information, such as majors and academic grades. In addition, we gathered course-related learning data from the Chaoxing Xuexitong system, a widely used online education platform in China. To obtain data on self-study hours, learning attitudes, and self-directed learning indicators, we employed questionnaires as the methodological instrument. The learning attitude questionnaire adapted from the English-learning Motivation Scale developed by a Chinese scholar Meihua Liu [40] who is from Tsinghua University, a tool commonly utilized in in EFL teaching and learning in the Chinese context. For assessing self-directed learning capabilities, we used a questionnaire adapted from Jinfen Xu ‘s [41] self-directed learning capability scale. These questionnaires were administered in class under instructor supervision and lasted approximately 10 minutes each, aiming to evaluate students’ learning attitudes and their aptitude for independent learning. The surveys were conducted midway through each semester. Our dataset encompasses data from 87 students enrolled in the Japanese course for the class of 2021, spanning three different learning modes. It includes nine indicators linked to student grades, amounting to a total of 2349 data entries. Table 3 shows the types of nine indicators.

**Table 3:** Description of indicator types.

Indicators	Indicator Type
**Classroom Performance**	Continuous
**Accuracy and Completeness of Responses in Class**	Continuous
**Absences from Class**	Continuous
**Semester Quiz Scores**	Continuous
**Time devoted to Self-study**	Continuous
**Attitude towards Learning**	Continuous
**Self-directed Learning capability**	Continuous
**Major**	Discrete
**Grade from the previous exam**	Continuous

While analyzing the datasets, an imbalanced data pattern was noted. To address this, we grouped students into three broad specialty categories: Arts, Science and Technology, and Arts and Sports. This categorization reduced data sparsity by assigning discrete values (1, 2, 3) to these groups.

### Ethical considerations

The study was approved by the institutional review board, and the study runs from September 2021 to June 2023. All participants were not at risk if they chose or declined to participate. Parental consent is not required for undergraduate students participating in the study. Additionally, we explained the purpose of the study in the questionnaire, clarified that it was their right to participate or not to participate in the study, and informed all the participants that ‘submitting answers’ is considered informed consent for researchers to use their questionnaire responses and related data retrieved from EAS and Chaoxing platform in publications of the research.

### Experimental setup

In this study, we conducted experiments employed PyCharm version 2022.3.3 as the compilation software, and implemented the algorithmic model using Python. The dataset was randomly partitioned into training and test sets in a 4:1 ratio for robust training and evaluation.

As state in the Methodology Section, we employ four classic ML models as our predictive model for academic performance. Table 4 presents the pseudo-code outlining the experimental procedures.

**Table 4:** The pseudo-code for the XGBoost-Shap.

**Algorithm 1:** XGBoost-SHAP.
**Input:** D represents the dataset containing all samples, *test*_*per* is the percentage of test data to the total number of D.M represents the predictive model, and N represents the number of training epochs
**Output:** importance_scores_set refers to the collection of feature scores for each test sample, where these scores indicate the importance of the features.
importance_scores_set = *ϕ*
D′ = feature_normal_and_mapping(D) // Continuous features are normalised
*D*_*train*_*D*_*test*_ = train_test_split(D, test_per)
for epoch = 0 to *N* do
ℳ = model_update(ℳ,*D*_*train*_)
for $x\in D_{test}$ do
importance_scores = SHAP(ℳ,*D*_*train*_)
importance_scores_set = importance_scores_set ∪ {importance_scores}

## Results

### Comparison of models

To obtain the optimal model parameters, the hyperparameters of the aforementioned four models were optimized separately. Table 5 displays the optimal hyperparameter combinations for the aforementioned four models.

**Table 5:** Optimal hyperparameter combinations of the four models.

Algorithms	Hyperparameter combinations	Optimal hyperparameter combination
**SVM**	C = (0.1,1,10,100) gamma = (0.0001,0.001,0.01,0.1,1)	C = 1 gamma = 0.001
**BPNN**	batch_size: = (8,16,32,64) lr = (0.00001,0.00004,by 0.000002) epoch = (500,800,1000,1500) min_epoch = (100,150,200) patience = (25,50,75) dropout_probability = (0.1,0.2,0.3)	batch_size: = 8 lr = 0.000038 epoch = 1000 min_epoch = 200 patience = 50 dropout_probability = 0.2 torch.optim.Adam
**RF**	n_estimators = (50,200, by = 10) max_depth = (20,50, by = 5) min_samples_leaf = (1,2,3,4,5,6) min_samples_split = (1,2,3,4)	n_estimators = 100 max_depth = 22 min_samples_leaf = 1 min_samples_split = 2
**XGBoost**	n_estimators = (100,1000,by = 100) max_depth = (3,20,by = 1) min_child_weight = (1,2,3,4,5) gamma = (0,0.1,0.2,0.3,0.4,0.5) subsample = (0.5,0.6,0.7,0.8,0.9,1) colsample_bytree = (0.5,0.6,0.7,0.8,0.9,1) reg_alpha = (0,0.1,1,2,3) reg_lambda = (0,0.1,1,2,3)	n_estimators = 100 max_depth = 6 min_child_weight = 1 gamma = 0 subsample = 1 colsample_bytree = 1 reg_alpha = 1 reg_lambda = 0

Table 6 presents the comparison of the task performance of four models. Both BPNN and XGBoost show higher task performance compared to RF, while SVM lags in terms of task performance. The comparison indicates that XGBoost slightly surpasses BPNN, establishing XGBoost as the model with the best predictive performance. Therefore, this study selects the XGBoost model to fit all the data. SHAP values are used for interpretation.

**Table 6:** Comparison of the ability to predict and fit of the four models.

Arithmetic	Offline teaching		Online teaching		Blended teaching	
	MAE	*R* ^2^	MAE	*R* ^2^	MAE	*R* ^2^
RF	7.8333	0.6617	5.2094	0.7202	6.8144	0.7475
BPNN	7.6987	0.7148	5.3880	0.7042	6.1815	0.8085
SVM	10.9029	0.2170	7.5030	0.4454	10.0406	0.4536
XGBoost	7.5578	0.7321	5.3710	0.7146	5.9176	0.816

### Exploratory analysis utilizing XGBoost and SHAP

Given the effectiveness of the XGBoost model, it was selected for further analysis using SHAP to explore teaching patterns within the model across various teaching modes. SHAP offers insights into the influence of each indicator per sample, highlighting both positive and negative effects. In the associated figures, color coding is used to represent the magnitude of eigenvalues, with red indicating high values and blue representing low values.

**Fig 2:**
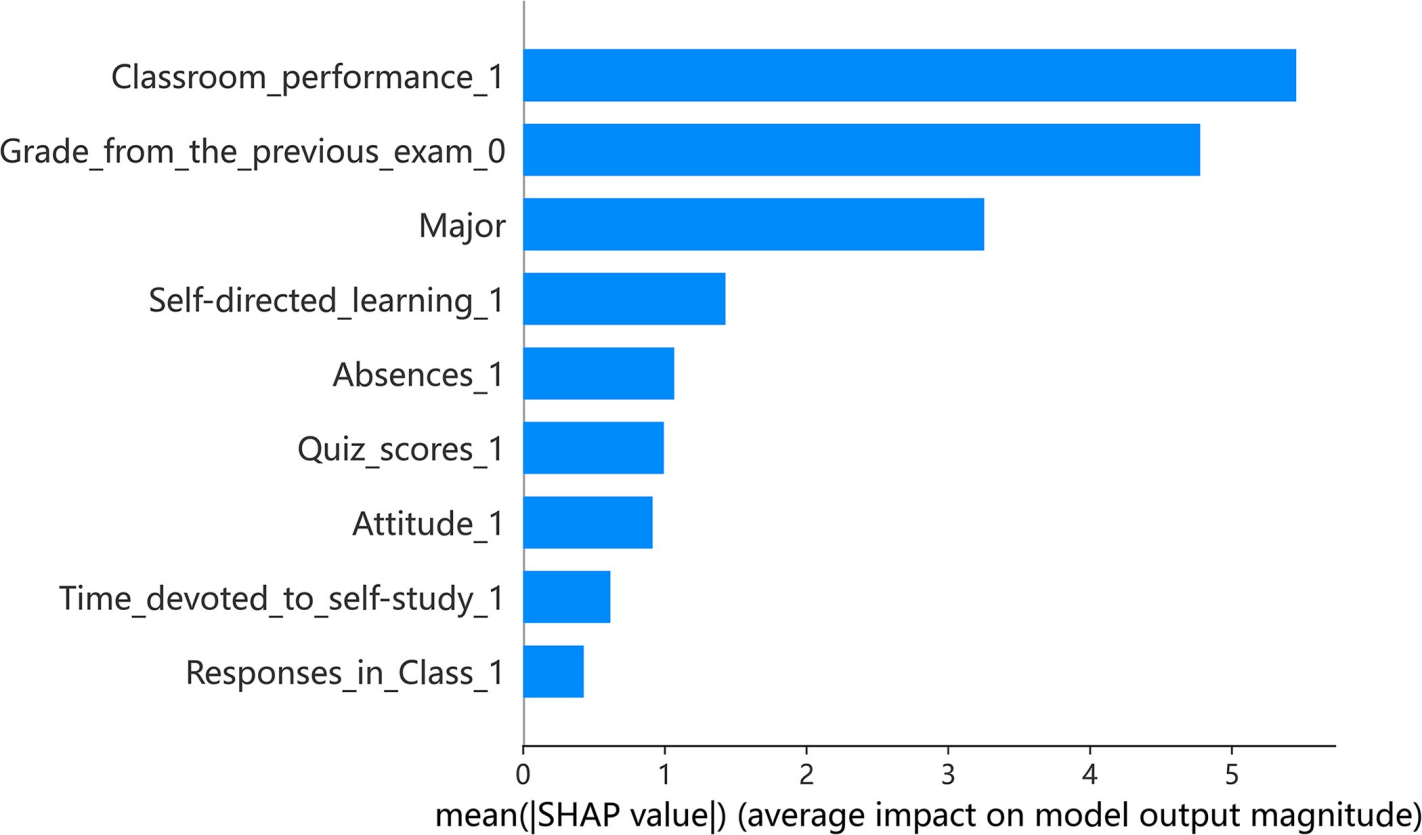
Indicator importance of offline teaching.

**Fig 3:**
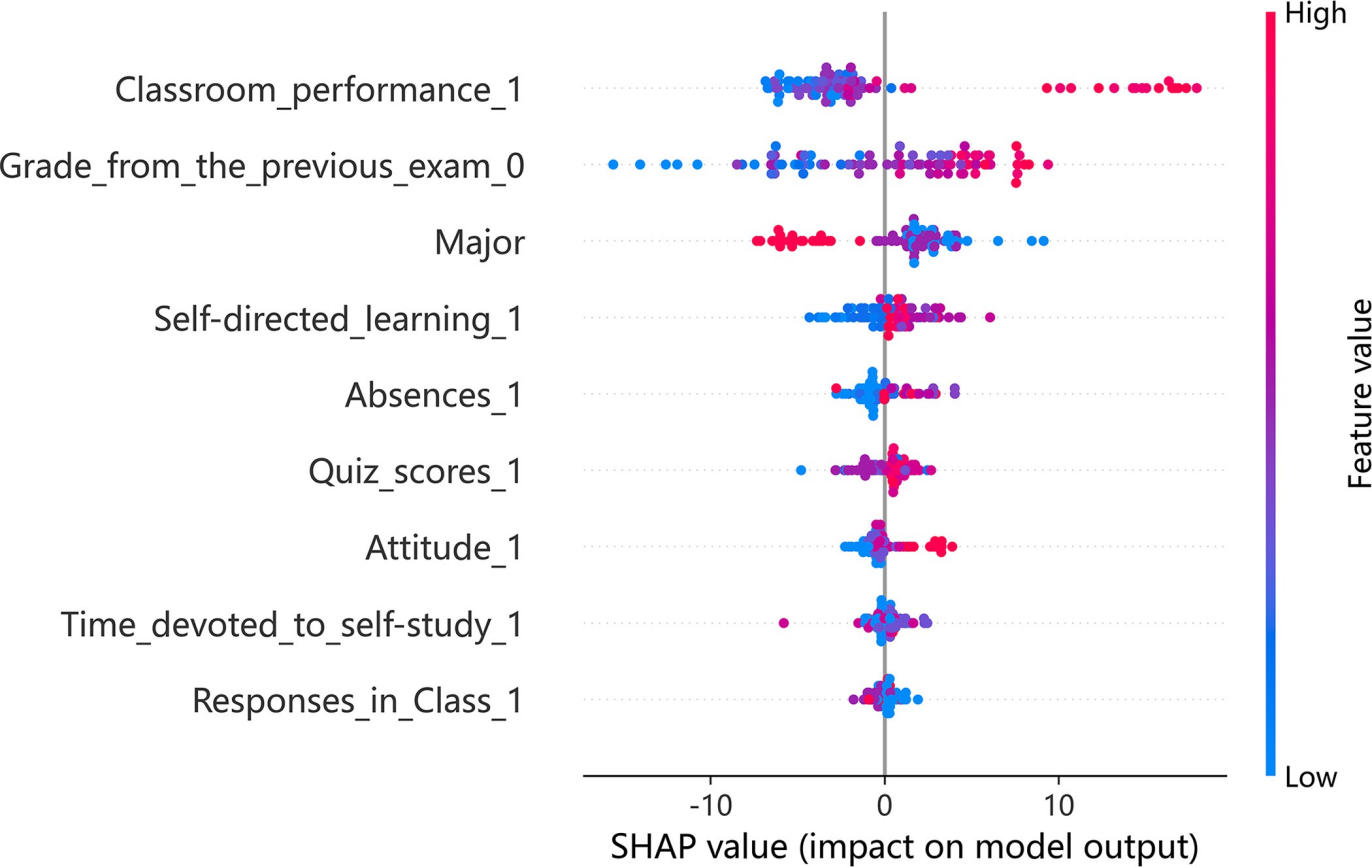
Summary plot of offline teaching.

Figs 2 and 3 shows the importance of indicators and a summary plot for offline teaching. The average SHAP value (horizontal axis) indicates the significance of each indicator, with their order of importance shown on the vertical axis in Fig 2. Key findings include classroom performance, previous exam grades, and student major as the most influential indicators. The impact of eigenvalues on each sample is depicted in Fig 3, where each row represents an indicator, each dot signifies a sample, and the SHAP value is plotted on the horizontal axis. Further analysis revealed a positive relationship between prior exam grades, self-directed learning ability, learning attitudes, and their effect on academic achievement predictions. Interestingly, occasional absences did not show a substantial negative influence on predicted grades, hinting at a divergence in the dynamics of college classrooms from high school settings. This might be attributed to the independent learning skills prevalent among college students. Moreover, it was noted that students majoring in Arts and Sports tend to have a slightly negative impact on predicted grades.

### Analysis of online teaching using XGBoost and SHAP

**Fig 4:**
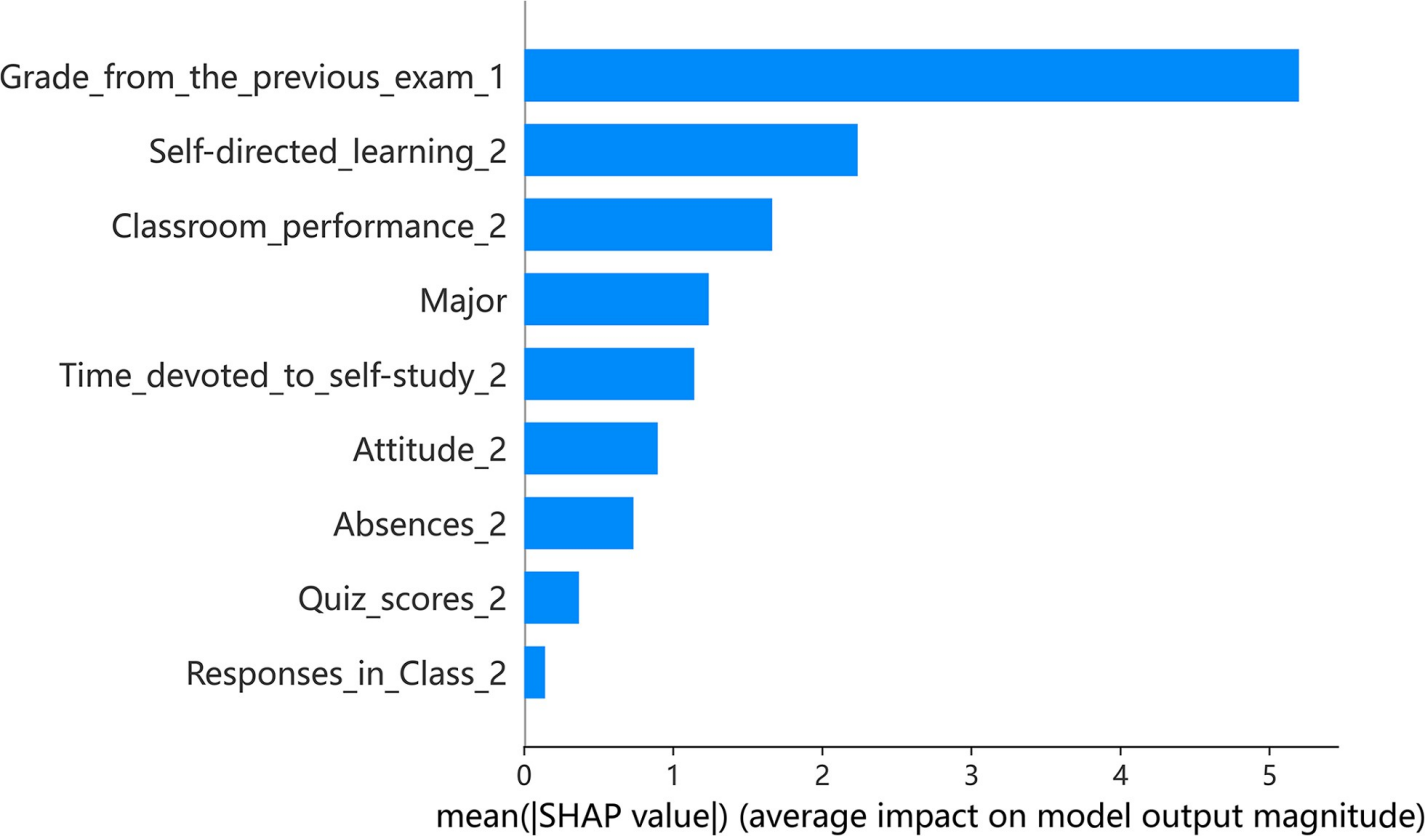
Indicator importance of online teaching.

**Fig 5:**
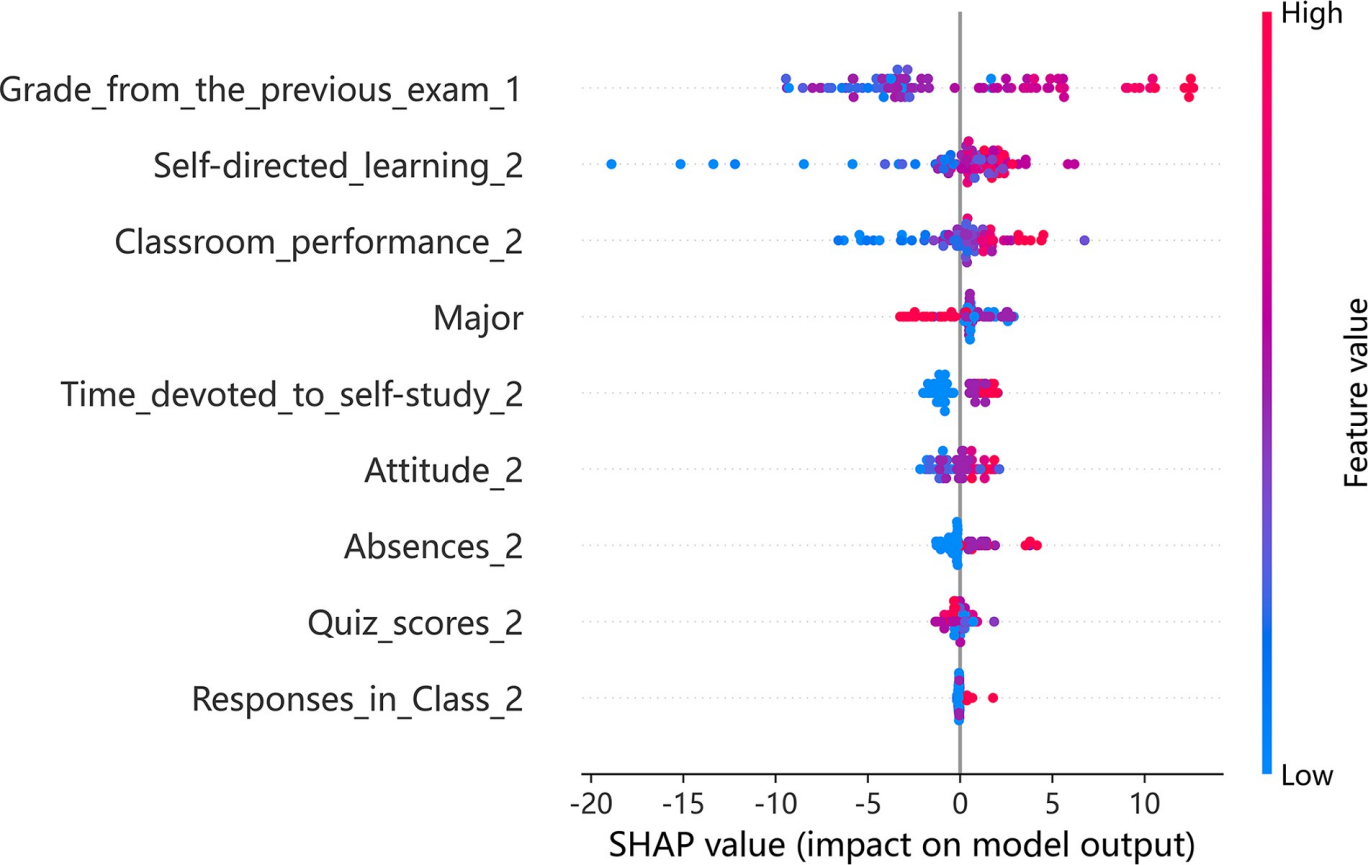
Summary plot of online teaching.

Figs 4 and 5 presents the indicator importance and summary plot for online teaching. A key observation is the increased influence of previous exam grades on the predicted values in comparison to offline settings. This suggests that students with a strong academic foundation tend to be more self-directed, thereby enhancing their predicted performance more remarkably. The disparity in self-directed learning abilities is more evident in online courses, highlighting the detrimental effect of inadequate self-learning skills on performance. Students struggling with self-learning might not receive timely support, leading to poorer outcomes. In this context, classroom performance becomes a less critical predictor, and the influence of a student’s major on predicted scores also diminishes. Interestingly, self-study time shows a positive correlation with predicted grades, while the relationship between quiz scores and performance prediction remains insignificant.

### Blended teaching: Insights from XGBoost and SHAP

**Fig 6:**
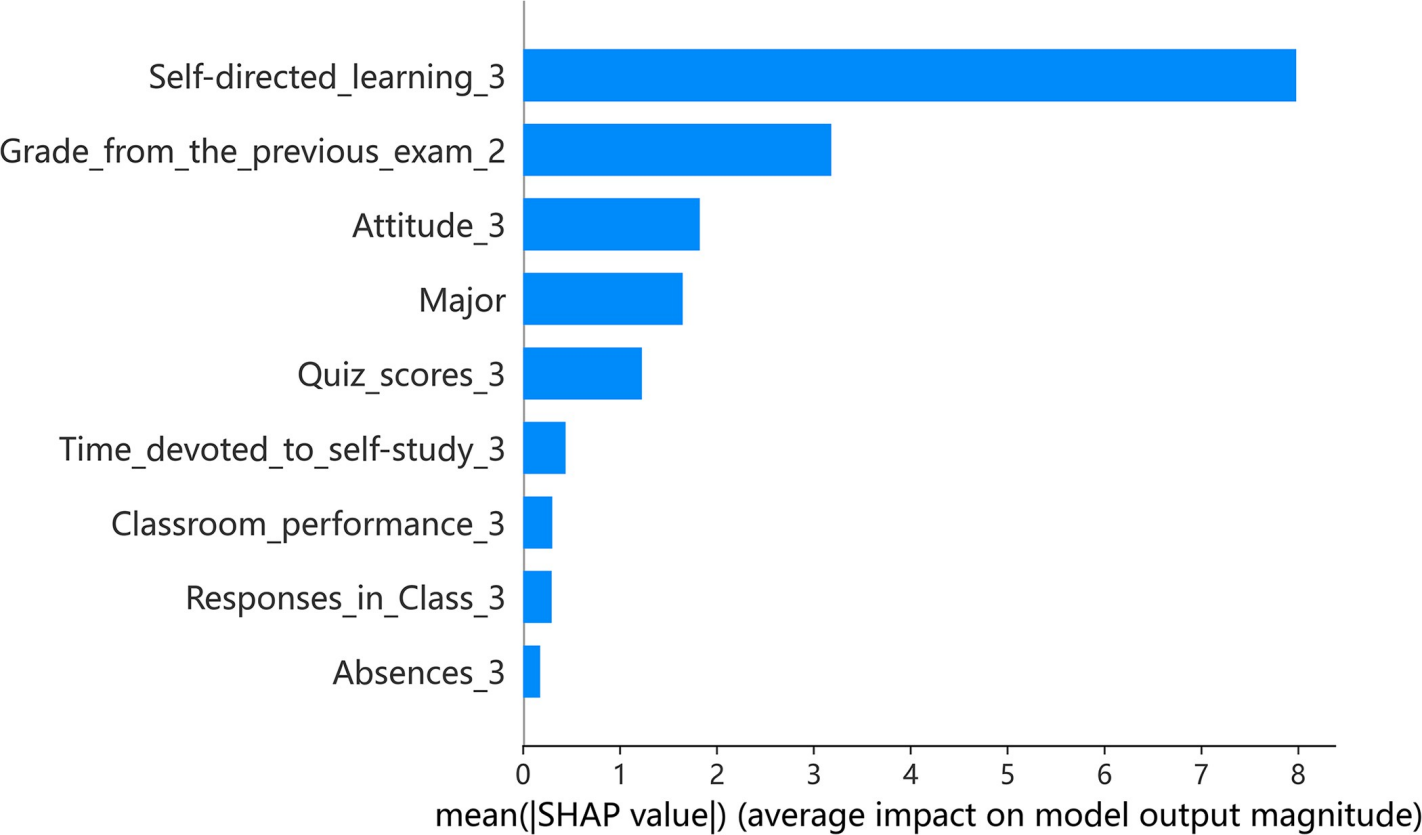
Indicator importance of blended teaching.

**Fig 7:**
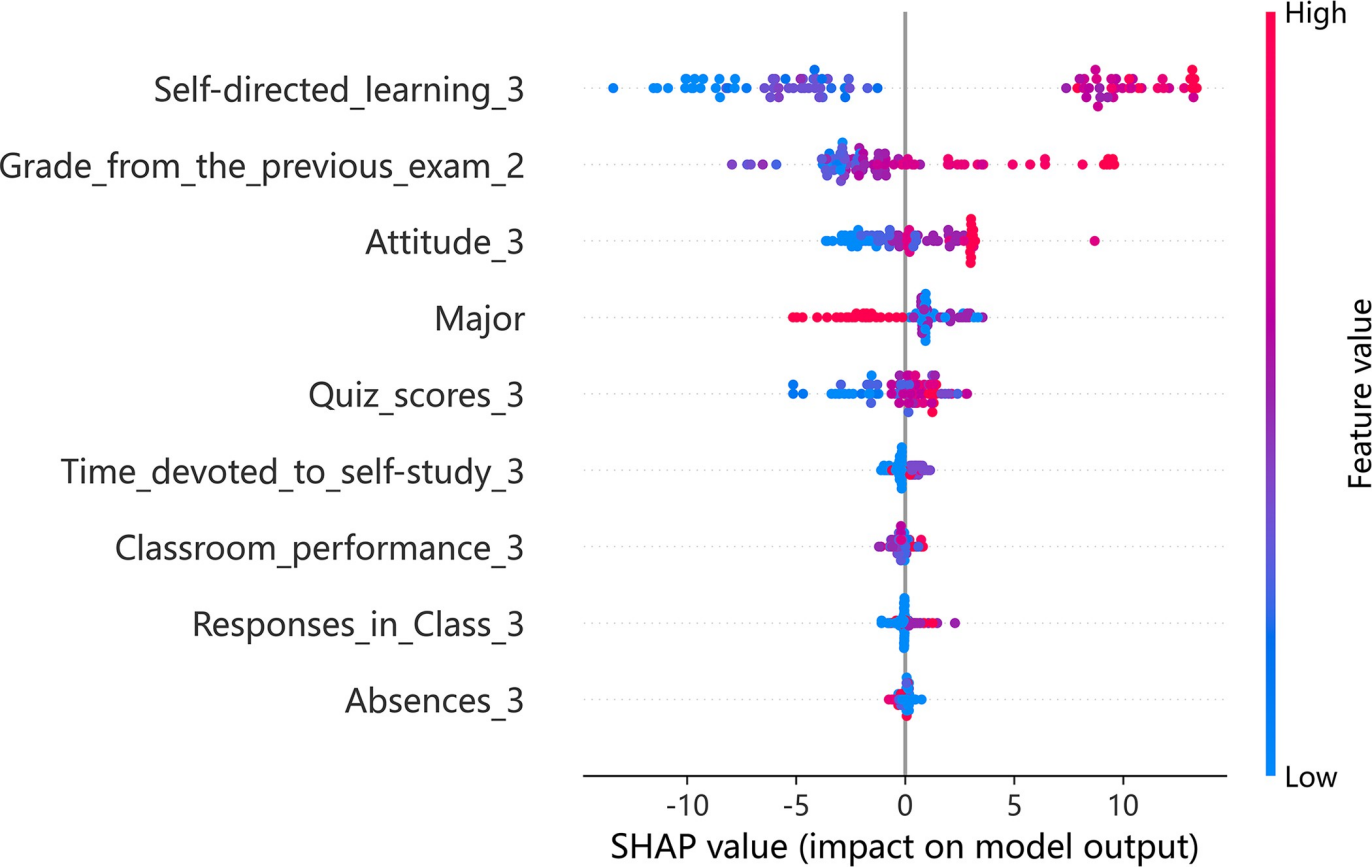
Summary plot of blended teaching.

Figs 6 and 7 examines the indicator importance and summary plot for blended teaching. In this teaching mode, the impact of self-directed learning skills is more notable compared to other teaching methods, possibly due to the adoption of flipped classroom techniques. Self-directed learning shows a stronger positive correlation with both previous exam grades and quiz scores. Furthermore, the relevance of attitude towards learning is accentuated, suggesting its growing importance in blended learning environments where independent study is emphasized.

### Discussion and conclusions

The prediction of academic achievement in higher education has become an increasingly prominent topic within the field of education [42]. In today’s information age, the tremendous growth of educational institutions’ electronic data “…can be utilized for discovering unknown patterns and trends” [43].Recent researches on predicting student performance are frequently spearheaded by educators identifying as "AI" educators to identify features that can be used to make predictions [44], to identify algorithms that can improve predictions [45], and to quantify aspects of student performance. However, analyzing performance, providing high-quality education strategies for evaluating the students’ performance from these abundant resources are among the prevailing challenges universities face [46].

In this research, we have developed the XGB-SHAP model, integrating XGBoost with SHAP, to systematically explore the relationship between grade prediction and diverse indicators across various teaching methods. Focused on university Japanese language classes, our study demonstrated XGBoost’s superior performance over other models, as evidenced by *R*^*2*^ and MAE metrics. The integration of SHAP offered a clear visual representation, highlighting the mode and directional influence of each indicator and sheds light on the educational implications of ML structures in pedagogy. The study also supported that the XGB-SHAP model can be effectively used in the field of educational management research.

The results reveal that, the study of student achievement prediction, using student-related features, such as student historical achievement, student engagement and demographic data, which have been used as important input features in the previous literature, is not sufficient. With the development of society and the diversification of teaching and learning modes, the importance of self-directed learning skills in the prediction of university students’ performance has been demonstrated in this study. Psychological factors such as attitude towards learning should also be taken into account. The impact of a student’s major on foreign language learning is considerable, which indicate differences in learning environments, cultural factors, motivation to learn foreign languages. While classroom response accuracy and attendance appeared less critical. This suggests a potential shift in focus within higher education classrooms, advocating for a tailored approach to characteristic selection based on teaching modes. This methodology provides educators with a quantitative view of how educational processes affect student achievement.

Our study also shows that the factors influencing student performance vary: offline teaching values classroom performance, while online teaching and blended teaching emphasize independent learning. In blended teaching, quiz scores have a remarkable positive impact, differing from the trends in other modes. This could be attributed to quizzes acting as formative assessments in blended learning, enhancing student participation and providing continual feedback. Consequently, teaching strategies and support systems should be adapted to meet the distinct needs of each teaching mode to optimize learning outcomes.

Acknowledging the formidable technical challenges associated with interpretable machine learning models in practical educational contexts, it is imperative to recognize their substantial contributions in enhancing our comprehension and utility of achievement prediction models. Additionally, they play a pivotal role in mitigating the skepticism harbored by educators towards machine learning models deployed for achievement prediction. Moving forward, there exist several promising avenues for exploration within the realm of interpretable machine models that merit thorough investigation: first, expand the dataset to cover more academic areas, different institutions, and varied student groups. This will test the model’s effectiveness in diverse settings. Second, the refinement and augmentation of existing interpretable models to enhance their accuracy and utility. These directions offer promising avenues for furthering the application and acceptance of interpretable machine learning in educational settings.

## Supporting information

S1 File Original data. (XLSX)
